# Success Rates of Assisted Reproduction for Men With Cystic Fibrosis

**DOI:** 10.1002/ppul.27472

**Published:** 2025-01-15

**Authors:** Karuna Sapru, Joanna Wilkinson, Anthony K Webb, Muhammad Akhtar, Rowland J Bright‐Thomas

**Affiliations:** ^1^ Department of Respiratory Medicine, Manchester Adult Cystic Fibrosis Centre, North West Lung Centre, Wythenshawe Hospital Manchester University NHS Foundation Trust Manchester UK; ^2^ Division of Immunology, Immunity to Infection & Respiratory Medicine University of Manchester Manchester UK; ^3^ Department of Reproductive Medicine and Surgery, St Mary's Hospital Manchester University NHS Foundation Trust Manchester UK

**Keywords:** assisted reproduction, azoospermia, cystic fibrosis, sperm retrieval

## Abstract

**Background:**

The vast majority of men with CF (mwCF) are infertile. Improvements in assisted reproductive technology (ART) have made it possible for these patients to become biological fathers.

**Methods:**

Data were examined for all male CF patients attending a large adult CF center over a 23‐year period. Azoospermia was confirmed via laboratory analysis of semen and was defined as complete absence of sperm in the ejaculate. Outcome of surgical sperm retrieval (SSR) procedure, post SSR complications, success rate of intracytoplasmic sperm injection (ICSI) and embryo transfer and subsequent live births were evaluated.

**Results:**

Seventy‐one mwCF with proven azoospermia were referred to fertility services over the study period. Mean (SD) percentage predicted forced expiratory volume in 1 second (ppFEV_1_) 67.86 ( ± 25.3), body mass index (BMI) 24.3 ( ± 3.7) kg/m^2^. Seventy (98.5%) of these men underwent and had successful SSR. 67/71 (94%) couples proceeded to have ICSI post SSR. 11 couples had failed egg fertilisation, implantation, or early miscarriage. 56/67 couples (84%) went on to have live births. To date 10/71 (14%) mwCF referred for fertility treatment have died. Mean ppFEV_1_ and BMI of mwCF who survived was higher than those who died; FEV_1_ 72.8% ( ± 23.4) versus 38% ( ± 12.0) (*p* < 0.001), BMI 25.0 ( ± 3.5) kg/m^2^ versus 20.3 ( ± 2.2) kg/mg^2^ (*p* < 0.001).

**Conclusion:**

This is the largest study to investigate success rate of fertility treatment in mwCF and demonstrates that fertility treatment is successful in greater than 75% of patients referred. Clinical status and prognosis remain important factors when considering referral to fertility services.

## Summary

- Study Question
  What is the current success rate, measured as live birth rate from mwCF with azoospermia who have been referred for specialist fertility treatment?
- Results
  Study demonstrates that success rates of assisted reproduction technologies for mwCF are excellent with 79% of patients referred for fertility treatment successfully becoming biological parents. Results are much better in this patient cohort than reported in other groups.
- Interpretation
  Prognosis continues to improve for CF patients. Assisted reproductive technologies have removed many limitations to parenthood for CF males.

**Abbreviations**
ART: Assisted reproductive technology
BMI: Body mass index
CF: Cystic Fibrosis
CBAVD: Congenital bilateral absence of vas deferens
ICSI: Intracytoplasmic sperm injection
IVF: In vitro fertilisation
MACFC: Manchester Adult Cystic Fibrosis Centre
MESA: Microepididymal sperm aspiration
mwCF: Men with CF
PESA: Percutaneous epidydimal sperm aspiration
PI: Pancreatic insufficiency
pwCF: Patients with CF
ppFEV1: Percentage predicted forced expiratory volume in 1 second
SSR: Surgical sperm retrieval
TESA: Testicular sperm aspiration
TESE: Testicular sperm extraction

## 1 Introduction

Cystic fibrosis (CF) is the commonest lethal autosomal recessive inherited condition affecting the western world [1]. It affects over 10,000 patients in UK and 35,000 patients in Europe [2]. Improved survival and health outcomes in CF patients have resulted in more men with CF (mwCF) and their partners considering parenthood. Approximately 98% of mwCF have congenital bilateral absence of vas deferens (CBAVD) and are infertile due to azoospermia [3]. Advancements in assisted reproductive technology (ART), including surgical sperm retrieval (SSR) and intracytoplasmic sperm injection (ICSI) before embryo transfer, has meant that mwCF can achieve biological paternity. The aim of this study was to examine data of mwCF referred for ART from the Manchester Adult CF Centre (MACFC) between 2000 and 2022, to determine the success rate of SSR, partner pregnancy rate and subsequent successful live births.

## 2 Methods

Data were examined for all mwCF attending MACFC between January 1, 2000 and December 31, 2022 that were referred for specialist fertility treatment. A prospective database was kept of all patients referred; results data were extracted from case notes and electronic patient records using a customised data collection form; all data were cross referenced with data from fertility services. These data were analysed on an anonymised secure database using Microsoft excel. All patients had a confirmed diagnosis of cystic fibrosis based on clinical presentation, CF genetic mutation analysis or sweat pilocarpine iontophoresis. Patient data and demographics including age, height, weight, percentage predicted forced expiratory volume in 1 second (ppFEV_1_), body mass index (BMI), and exocrine pancreatic status were collected. Azoospermia was confirmed via laboratory analysis of semen and was defined as complete absence of sperm in the ejaculate [4]. Data were analysed on outcome of SSR procedure, post SSR complications, success rate of ICSI and egg fertilisation, embryo transfer and subsequent live births.

Statistical analysis was performed using IBM® SPSS® Statistics version 27. Chi‐square tests were used to examine ART outcomes data between patient subgroups. Unpaired T‐tests were used to examine differences between patients who died over the study period versus those who survived in respect of lung function, weight, and BMI. The conventional 0.05 level of significance was used. Numerical data is presented as mean (standard deviation) unless otherwise stated. Study reviewed by research and development office; research ethics committee approval and informed consent was not required for this retrospective service evaluation conducted via analysis of existing data with no change in treatment, patient intervention or patient identifiable data.

## 3 Results

Over the 23‐year study period, 71 mwCF were referred to regional fertility services from MACFC. Demographic data is shown in Table 1. All mwCF referred for fertility treatment were aged over 18, were counseled by CF team and had proven azoospermia on testing both before referral and repeated by fertility service. Mean (SD) age of mwCF at the time of SSR was 31.3 (±4.6) years. Fifty eight (82%) were pancreatic insufficient and 13 (18%) patients pancreatic sufficient. Mean ppFEV_1_ of mwCF at the time of referral to fertility services was 67.86 (±25.3) % and BMI was 24.3 (±3.7) kg/m^2^. All 71 mwCF referred to fertility services underwent SSR procedure which was successful with viable sperm recovered in 70/71 (98.6%). There were no serious surgical or anesthetic complications post procedure including bleeding or local infection, but postoperative pain was frequently reported, one patient required subsequent urological referral for pain and was diagnosed with epididymal cyst and three patients required treatment with intravenous antibiotics for treatment of an infective exacerbation of CF within 2 weeks of the procedure.

**TABLE 1:** Demographic data of men with CF at time of SSR.

Referred to fertility services	71	
Age, years ( ± SD)	31.3 (4.6)	
Exocrine pancreatic insufficiency (%)	58 (82%)	
FEV_1_ percent predicted (%) ( ± SD)	67.86 (25.3)	
BMI (kg/m2) ( ± SD)	24.3 (3.7)	
**Patient outcome**		
Alive (n = 61)	Age	31.0 (23‐41) years
	ppFEV_1_	72.8 (±23.4) %
	BMI	25.0 (±3.5) kg/m^2^
Dead (n=10)	Age	33.3 (25‐39) years
	ppFEV_1_	38 (±12.0) %
	BMI	20.3 (±2.2) kg/m^2^

**FIGURE 1:**
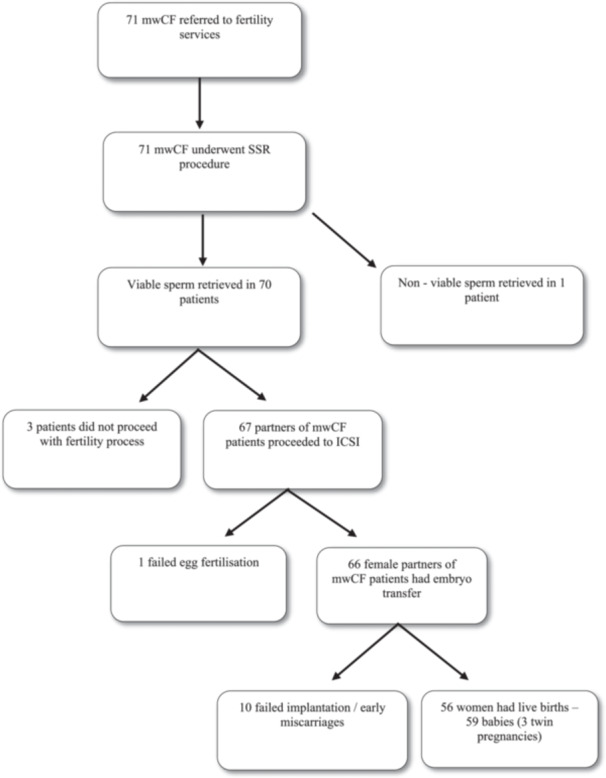
**Outcome of fertility service referral for men with CF**.

3/71 (4%) patients did not proceed with the fertility program following sperm retrieval; one due to personal patient preference, one patient died before his wife progressed to ICSI and one patient had elective SSR and sperm banking before commencing chemotoxic treatment before renal transplantation. 67/71 (94%) patients went on to have ICSI post SSR. One couple had failed egg fertilization, presumed due to poor sperm motility, but later achieved a successful pregnancy via donor sperm insemination. Ten further couples with successful egg fertilisation had embryo transfer but subsequent failure of implantation, or early miscarriage. 56/67 couples (84%) achieved pregnancy and went on to have live births with no babies having CF, congenital or chromosomal abnormalities. Figure 1 below highlights the outcome of all 71 men referred to fertility services.

Data grouping patients according to CF genotype, modulator treatment taken, age and time of SSR is shown in Table 2. No difference in ART success rate was observed between different genotype or modulator treatment groups. On examination of patient age at time of SSR overall success rates were ≥ 80% for all groups except patents aged ≥ 40 years where success rate 0%. This was a significant difference (*p* = 0.029) but may be an anomalous result as only two patients were in the ≥ 40 (both had successful SSR with viable sperm retrieved and subsequent oocyte fertilisation but failure of implantation). Additionally no difference in success rate was observed between lung function or BMI range and there was no difference between pancreatic sufficient and insufficient patients. When analysed in ART date cohorts increasing patient referral numbers and treatment was observed over time (lower number 20–23 due to COVID‐19 pandemic), but no trend was identified for improved success rates during the time period analysed.

**TABLE 2:** ART outcome data comparing mwCF genotype, modulator status, age and date of SSR.

	Live births	Failed ART	Did not proceed post SSR	ART success rate (%)
**Genotype**				
F508del/F508del (*n* = 30)	24	5	1	83%
F508del/other (*n* = 34)	28	5	1	85%
other/other (*n* = 7)	4	2	1	67%
**CFTR modulator therapy**				
None (*n* = 59)	46	11	2	81%
Ivacaftor (*n* = 6)	4	1	1	80%
Lumacaftor/Ivacaftor (*n* = 2)	2	0	0	100%
Elexacaftor/Tezacaftor/Ivacaftor (*n* = 4)	4	0	0	100%
**CF patient age at time of SSTR**				
Age 20–24 (*n* = 3)	2	0	1	100%
Age 25–29 (*n* = 22)	17	4	1	81%
Age 30–34 (*n* = 29)	25	3	1	89%
Age 35–39 (*n* = 15)	12	3	0	80%
Age ≥ 40 (*n* = 2)	0	2	0	0%*
**Date cohort of SSR**				
2000–2004 (*n* = 3)	3	0	0	100%
2005–2009 (*n* = 9)	6	3	0	67%
2010–2014 (*n* = 32)	26	5	1	84%
2015–2019 (*n* = 22)	16	4	2	80%
2020–2023 (*n* = 5)	5	0	0	100%

Fertility assessments were performed in all patients by Reproductive Medicine Department. Complete hormone profiles were available in 61/71 patients. Mean testosterone 15.7 nmol/L (normal range 10‐50), follicle stimulating hormone (FSH) 6.1 IU/L (1.5–12.4), luteinising hormone (LH) 5.2 IU/L (1.7–8.6). Results of testicular ultrasound were available in 55/71 patients. 36/55 were reported as entirely normal but tubal and ductal ectasia, atrophic and hypoplastic epididymi, epididymal cysts, microlithiasis (two patients) and varicoceles were also reported. Complete data on SSR procedure and anesthetic performed was available in 53/71 patients. Thirty‐two patients had testicular sperm extraction (TESE) and 23 patients percutaneous epidydimal sperm aspiration (PESA), two patients had both procedures. SSR was performed with general anesthetic in 24 patients, sedation and local anesthetic in 21 patients, and performed under spinal anesthetic in 8 patients. The one patient who did not have viable sperm recovered by SSR had PESA under sedation. Testicular ultrasound reported that testes were heterogeneous in texture, with left testes smaller than right and right sided ductal ectasia. Poor spermatogenesis was presumed related to use of gym hormone and protein supplements in this patient.

To date 10/71 (14%) mwCF referred for fertility treatment over study period have died. Five of these patients died within 2 years of birth of their child. The mean (SD) ppFEV_1_ of mwCF who survived was 72.8% (±23.4) versus 38% (±12.0) predicted in those who died (*p* < 0.001). BMI of patients who survived was higher, 25.0 (±3.5) kg/m^2^ than patients who died 20.3 (±2.2) kg/mg^2^ (*p* < 0.001). Table 1 outlines the demographic data of the men involved at the time that they were referred for fertility input.

## 4 Discussion

As the prognosis for people with CF improves, having a family of their own is becoming increasingly common. This is the largest study to date of fertility success rates in mwCF. Seventy nine percent of men referred to fertility services went on to become biological parents demonstrating that assisted reproductive technologies have removed many of the limitations to parenthood in this patient group.

CF is a multisystem disease resulting from mutations in the gene which encodes the CF transmembrane conductance regulator (CFTR) protein, a complex chloride channel involved in producing sweat, mucous, tears and digestive enzymes [5]. Registry data demonstrates that the median predicted survival of people with CF has increased to 64.1 years in the UK [6]. This is as a result of a combination of improvements in diagnosis, specialist multidisciplinary team delivered care and therapeutic options. Despite extended life expectancy the disease burden in CF remains high and living with CF has become increasingly complex.

Increased longevity and improved quality of life has resulted in an increasing number of mwCF expressing their wish to have children. Around 98% of men with CF have congenital bilateral absence of vas deferens (CBAVD) resulting in obstructive azoospermia which precludes natural conception [3]. Men with absence of vas deferens tend to have acidic low volume semen and infertility may be the first presentation in adulthood of CF gene dysfunction [7]. At MACFC all patients requesting referral to fertility services are counseled before referral. This includes discussion regarding patient prognosis and potential implications of pregnancy and parenthood, screening of partners for CF gene mutations and confirming azoospermia by semen analysis [8] collected 3–5 days after abstinence which is repeated by fertility services. During the twenty year study period only 3 mwCF attending MACFC were identified as having motile sperm and conceived naturally, however exact number is unknown as patients not requesting fertility input did not receive semen analysis.

Advancements in ART techniques to harvest and inject sperm directly into oocytes in vitro has allowed CF men to achieve biological parenthood. Sperm from proximal caput epididymis or from the intratesticular vasa efferentia can fertilize a human oocyte in vitro and result in successful pregnancy and subsequent live birth [9]. Sperm can be retrieved from the epididymis by direct aspiration via PESA (percutaneous epidydimal sperm aspiration) or surgically via MESA (microepididymal sperm aspiration). PESA is a less invasive procedure which requires no incision and has a quick recovery time which can be performed in an outpatient setting under local anesthetic or sedation; it can provide mature sperm but is unlikely to be successful when the epididymis is hypoplastic. MESA usually requires a general anesthetic as epididymis is dissected before fluid from epididymal tubule is aspirated. Testicular procedures, testicular sperm aspiration (TESA) and testicular sperm extraction (TESE), provide less mature sperm. TESE requires a small incision in the scrotal skin with biopsies taken from the testicular tissue and is more likely to result in post operative pain [10]. Scrotal ultrasound and testicular examination may be helpful in deciding which technique offers the best chances of success for the individual patient.

In vitro fertilisation (IVF) usually involves incubating 100–150,000 sperm with oocytes and waiting for fertilization to take place when one sperm enters the oocyte. Intracytoplasmic sperm injection (ICSI) was introduced in 1992 as an IVF advancement and used primarily for the treatment of male‐factor infertility. In this technique, following female ovarian stimulation and oocyte retrieval, the surrounding cumulus of cells from the oocyte is removed and a single selected sperm is directly injected into the cytoplasm of the oocyte. The necessity of only one viable sperm for fertilisation per mature oocyte provides the opportunity of parenthood for patients who have a reduced number of viable sperm [11]. If fertilisation takes place the developing embryo is cultured for 3–5 days before being transferred to the uterine cavity, this technique achieves the best outcome by bypassing all the barriers that the sperm would to have to overcome in natural conception. A small number of studies have raised concerns about the potential of pregnancies resulting from ICSI to have higher risk of congenital malformation and chromosome aneuploidy compared to children conceived naturally [12]. In our study all children born were healthy with no congenital or chromosomal malformations; this is consistent with other data that risk of chromosomal aberration following ICSI in CBAVD patients is no higher than that in normal fertile men [13].

A large non‐CF study estimating a couples' probability of having a live birth, combining IVF and ICSI procedures, demonstrated that 29% had a live birth after their first cycle of treatment and 43% had a successful live birth over six complete cycles of IVF or ICSI [14]. The US CF Foundation quote that success rates for people with CF having IVF are typically between 20% and 40% [15] but this relates to assisted reproduction in both males and females with CF where the causes of infertility is different. Our study examines success rates of ART in men with CF treated within UK NHS structure; other countries may have differences in ART services and provision. A previous retrospective French study in mwCF found that the fertilisation rate with ICSI was 61% although all fertilised eggs did not go on to form healthy embryos and pregnancy rate in the study was 40% per ICSI cycle [16]. A retrospective US study of mwCF by McCallum et al reports eight men with CF undergoing SSR and their partners undergoing one or more cycles of ICSI; five couples achieved pregnancy (62.5%) but only four couples delivered, giving an 50% success rate [17]. The data we present here are the largest published study to date of fertility success rates in mwCF and has a larger patient cohort size than both the aforementioned studies combined. The results demonstrate that assisted reproduction via SSR, ICSI and subsequent embryo transfer have very high success rates in CF adults. In this cohort SSR was successful with viable sperm recovered in 70 of 71 patients. One other patient was reported to have poor sperm motility and egg fertilization was unsuccessful; this couple proceeded to subsequent successful pregnancy using donor sperm insemination. Fifteen percent of couples had successful egg fertilisation and embryo transfer but subsequent fetal loss due to failed implantation or early miscarriage. Of the 71 mwCF who were referred to fertility services, 56 (79%) achieved successful live births with their partner. Fifty nine healthy babies were born to 56 couples who have subsequently had an additional 18 babies following further cycles with 15 couples having had more than one child. It has been reported in one study that homozygous F508del patients have worse SSR and ICSI outcomes [18] but in our study the ART success rate in both this group and heterozygous F508del group were greater than 80%. The majority of these data relate to fertility treatment before widespread introduction of CFTR modulators. No difference in outcomes according to modulator status was observed but these data need further clarification in future studies. These data are extremely encouraging for the male CF population. The higher success rate observed in mwCF compared to other patient cohorts reflects advances in reproductive technology combined with fact that primary reason for infertility in these patients was obstructive azoospermia rather than abnormal or absent sperm production or female factors.

MwCF are living longer healthier lives and proceed through reproductive milestones at a similar age to males in the general population. These patients have the additional challenges of chronic illness, complex treatment regimens and infertility which may make having and sustaining relationships difficult [19]. The longevity of any parent is not guaranteed but CF adults often have a high disease burden, intensive treatment regimen and life limiting disease which can restrict the time they have with their children [11]. In our study period 14% patients died after starting fertility treatment; these patients tended to be older with worse lung function and lower BMI. It is therefore a very realistic possibility that mwCF may not survive to see their children through to adulthood. CF centers should discuss health status and prognosis carefully with mwCF and their partners when considering parenthood, especially those CF patients with poor baseline parameters such as lung function and BMI.

Studies have shown that mwCF would prefer the subject of infertility to be introduced by health professionals as early as adolescence [20, 21]. Reproductive and genetic counseling should be offered to any CF patient and their partners if they are considering becoming a parent. At our Centre all couples who chose to proceed with fertility service referral received counseling with regard to patient clinical status, ART process and pre pregnancy screening of partners for CF gene variants. The risk of being a CF gene carrier is one in 25 in the Caucasian UK population. The couple need to be advised that carrier testing of partners cannot detect all potential CF mutations within the gene and that a negative screen significantly reduces the risk of the individual being a carrier but does not eliminate it completely [22]. Where the partner is found to be a CFTR mutation carrier, there is a possibility to perform pre‐implantation genetic diagnosis (PGD). In this process following SSR and ICSI embryos are tested for CF and only non‐affected embryos implanted thus preventing the birth of children affected with CF.

For couples who do not wish to go through the assisted reproduction process or where it has not been successful options include the use of donor sperm or adoption. At MACFC 4 of 11 couples who failed ICSI treatment went on to adopt children and one couple used donor insemination. At least another four men have adopted children and three couples used donor sperm during this study period having not requested referral to fertility services.

This study does have limitations. The CF patients studied all attended one adult CF Centre with complete data set of patients referred for SSR, subsequent ICSI and live birth success rates with none lost to follow‐up, however full data is not available on those patients who, post counseling by CF team, decided to not proceed with referral to fertility services referral or went on to explore adoption, use of donor sperm or surrogacy. Full data from fertility center, including fertility assessment, SSR procedure undertaken and postoperative pain reports, were not available for all mwCF and partner data including number of treatment cycles received and cause of fetal loss in 10 females post implantation is not known. Further studies are required to evaluate the impact of poor baseline parameters such as spirometry or nutritional status, pharmacological CF therapy and CF co‐morbidities on fertility treatment. Further work is also required to improve our understanding of the psychological as well as physical long‐term outcome for CF patients and their families when undergoing fertility treatment.

## 5 Conclusion

This is the largest study of male CF fertility success rates to date and demonstrates that fertility treatment results for mwCF are excellent, with 79% of patients referred for fertility treatment successfully becoming biological parents. CF Centres should, however, consider carrying out a thorough risk assessment of CF patients especially those with poor baseline weight and lung function and ensure they have frank discussions with patients and their partners about long term prognosis and disease management before referral to fertility services.

## Author Contributions

**Karuna Sapru:** Data curation; Formal analysis; Writing–review & editing; Writing–original draft. **Joanna Wilkinson:** Data curation; Formal analysis; Writing–original draft. **Anthony K Webb:** Conceptualization; Writing–review & editing. **Muhammad Akhtar:** Data curation; Writing–review & editing. **Rowland J Bright‐Thomas:** Conceptualization; Data curation; Supervision; Writing–review & editing.

## Conflicts of Interest

The authors declare no conflicts of interest.

## Data Availability

The data that support the findings of this study are available from the corresponding author upon reasonable request.
